# Hygienic safety of reusable tap water filters (Germlyser^®^) with an operating time of 4 or 8 weeks in a haematological oncology transplantation unit

**DOI:** 10.1186/1471-2334-7-45

**Published:** 2007-05-23

**Authors:** Georg Daeschlein, William H Krüger, Christian Selepko, Markus Rochow, Gottfried Dölken, Axel Kramer

**Affiliations:** 1 Institute for Hygiene and Environmental Medicine, Germany; 2 Dept. of Internal Medicine C – Haematology and Oncology, Stem Cell Transplantation, Ernst Moritz Arndt University, Greifswald, Germany

## Abstract

**Background:**

Microbial safe tap water is crucial for the safety of immunosuppressed patients.

**Methods:**

To evaluate the suitability of new, reusable point-of-use filters (Germlyser^®^, Aquafree GmbH, Hamburg, Germany), three variations of a reusable filter with the same filter principle but with different outlets (with and without silver) and inner surface coating of the filter encasements (with and without nano-crystalline silver) were tested. The filter efficacy was monitored over 1, 4 and 8 weeks operating time in a haematological oncology transplantation unit equipped with 18 water outlets (12 taps, 6 showers).

**Results:**

The filtered water fulfilled the requirements of absence of pathogens over time. From 348 samples, 8 samples (2.3%) exceeded 100 cfu/ml (no sample ≥ 500 cfu/ml). As no reprocessed filter exhibited 100% filter efficacy in the final quality control after each reprocessing, these contaminations could be explained by retrograde contamination during use.

**Conclusion:**

As a consequence of the study, the manufacturer recommends changing filters after 4 weeks in high risk areas and after 8 weeks in moderate infectious risk areas, together with routine weekly alcohol-based surface disinfection and additionally in case of visible contamination. The filter efficacy of the 3 filters types did not differ significantly regarding total bacterial counts. Manual reprocessing proved to be insufficient. Using a validated reprocessing in a washer/disinfector with alkaline, acid treatment and thermic disinfection, the filters were effectively reprocessable and now provide tap water meeting the German drinking water regulations as well as the WHO guidelines, including absence of pathogens.

## Background

Worldwide, nosocomial waterborne pathogens play an important and underestimated role in infection [1,2]. Especially in the last decade, water taps as an origin of infection were identified by epidemiological and molecular methods [1,3]. Substantial reservoirs for potential pathogens are tap water and siphons [4,5]. The most common water pathogens are *Legionella pneumophila, Pseudomonas aeruginosa *and moulds [6]; they are set free as planktonic contaminants from biofilm in the water supply [7]. Typical transmission pathways of water-associated bacteria are taking a shower, body washing, wound rinsing, washed hands, and the occurrence of water splashing in the ward [3]. Heavily immunosuppressed patients, e.g., after allogeneic stem cell transplantation, as well as intensive care patients are highly susceptible to waterborne nosocomial infections [8,9], and infection of immunocompromised hosts with *Pseudomonas spp*. or *Aspergillus spp*. are often fatal. Barrier nursing is the key to preventing nosocomial infections, both waterborne and other types [10-13]. One of its essential aspects consists in providing water free of pathogens, which can achieved by use of point-of-use (POU) filters or sterile bottled water [7,14,15]. The introduction of a new type of reprocessable POU filter with tubular ceramic filter surfaces (hollow fibre) instead of conventional single-use filters with flat fabric filters offers economical and ecological advantages in the field of water safety and resource management (sustainable development). For the filter types tested here, the manufacturer indicated filter reprocessing after one week's operating time. Based on both hygienic and economical concerns, the purpose of this study was to evaluate the safety of longer operating periods (4 and 8 weeks) in a bone marrow transplantation (BMT) unit.

## Methods

### Filters

Three consecutively developed modifications of a reusable filter (Germlyser^®^, Aquafree GmbH, Hamburg, Germany) were tested: filter type 1, hollow fibre filter of polyethersulfon with a pore size of 0.2 μm and a surface area of 800 cm^2^; filter type 2, the same hollow fibre filter but with 1100 cm^2 ^of filter surface and a nano silver coating of the encasement's inner wall; and filter type 3, the same as filter type 2 but with a metallic silver outlet. The shower filter (3000 cm^2^) possessed the same hollow fibre system with a surface area of 3000 cm^2^, and was coated with nano silver as in filter type 2.

### Design of the prospective in-use study

Between January 2005 and October 2006, we conducted a prospective in-use monitoring of filter efficacy by testing the microbial water quality – according to German drinking water regulations and the WHO guidelines for drinking-water quality [2,16] – in the haematological oncology transplantation unit of the University Hospital of Greifswald, Germany. The unit includes 6 single-patient rooms with bathrooms, with each room separated from the corridor by an individual ante-room. Each ante-room and bathroom is equipped with a washbasin, and each bathroom additionally with a shower. In addition, all patient rooms have HEPA-filtrated air-conditioning to avoid airborne infections such as pulmonary aspergillosis. All water outlets and showers (in total: 12 tap water outlets and 6 showers) were provided with the POU filters and tested for microbial water quality.

The study included 4 trials (table 1). In the first trial, the water contamination was tested with a filter operation time (filter type 1) of one week combined with manual reprocessing. In manual reprocessing, the filters were flushed directly at their faucet over 30 sec, followed by heating in a 95°C water bath for 10 min. Each reprocessing was controlled by a manual leakage test with compressed sterile air according to DIN 58356-2 [17]. In this trial only, we sampled sludge from the inner (proximal) filter surface after opening the filter screw.

**Table 1:** Tested filter types, operating time and test intervals (trial 1–4)

**Filter type/trial**	**sample sets (n samples)**	**Operating time of filter (weeks)**	**Additional test intervals during operating time (weeks)**
1/1	3 × 6 (18)*	1	0
1/2	13 × 6 (78)*	1	0
2/3	12 × 18 (216)	4	1
3/4	3 × 18 (54)	8	1, 4

* without showers

In trial 2, filter type 1 was also used over 7 days, but filters were automatically reprocessed in a washer/disinfector (E 7736CD, Miele GmbH, Spreitenbach, Germany) at the certified Central Sterile Supply Department (CSSD) of the University hospital of Greifswald (process parameters in table 2). After reprocessing, the filters were tested for leakage in the same way as described above and stored in sterile boxes.

**Table 2:** Workflow of reprocessing in the washer/disinfector and further processing

Step	Parameters
Flushing	25°C, 2 min, RO (reverse osmosis) water
Basic cleaning and disinfection	(NaOH) pH 11, RO water, 50°C/5 min
Rinsing	RO water 25°C/1 min
Acidic cleaning to remove mineral stains of calcium carbonate	phosphoric acid, pH 2, RO water, 50°C/5 min
Final rinsing	RO water 25°C/1 min
Thermic disinfection	95°C/10 min, RO water
Check for membrane integrity	resistance time over 1 min
Drying with sterile air	115°C
Packing and documentation	sterile boxes

For trial 3, the filters of type 2 and for trial 4 the filters of type 3 were used for 4 and 8 weeks, respectively. The outsourced reprocessing was conducted by Aqua free Membrane Technology in Hamburg, Germany. Aqua free is certified according to EN ISO 13485 (2003) and the filters are reprocessed automatically in a washer/disinfector (Miele 7736 CD), tested according to DIN 58356-2 and dried at 115°C with sterile filtrated air.

In trials 1–4, we analysed unfiltered water samples in parallel to each sample set as control for the bacteriological load of the unfiltered water.

Except trial 1 and 2 (sample sets of 6 tap water filter samples in the bathroom of patients), a sample set consisted of 18 samples (12 tap water + 6 showers). The samples were taken directly before changing the filters. During trial 1, they were taken once a week (after 3 weeks, the trial was discontinued), and in trial 2 with 13 sample sets, samples were initially taken once a week for 7 weeks, and thereafter once a month for 6 months (31 weeks). In trial 3 with 4 weeks of operating time, the samples were taken weekly for 3 months (12 sample sets). In trial 4 with 8 weeks of operating time, samples were taken after the first week, then after 4 weeks and finally after 8 weeks (1 sample set each time).

### Sampling

The water quality was approved according to the German Drinking Water Guidelines [16]. Immediate samples of 350 ml of cold and a second portion of 1000 ml of hand-warm, mixed cold and hot tap water were taken at each sampling. In accordance with Pitten et al., we did not apply flame to the outlets of filtered and unfiltered taps, in order to simulate real risk conditions [18]. During trial 3 and 4, the filters were disinfected weekly on a fixed day by wiping the filter encasement and outlet with 70% propan-2-ol, which had not been recommended by the manufacturer.

### Microbiological analysis

Total bacterial content was determined by direct cultivation (semi-solid medium method after Koch) at 22°C and 36°C in parallel with incubation for 48 h and visual counting. To determine whether testing at only one temperature (22°C or 36°C) yields the same results, we compared the sensitivity (number of cfu) of the two culture temperatures during the longest test period (trial 2) and decided to test solely at 22°C in trial 3. Because of the highest risk of retrograde contamination in trial 4 (longest operation time), we tested the filter efficacy again with two cultivation temperatures.

To detect *Coli-like*, *P. aeruginosa *and faecal enterococci, 3 × 100 ml of water were filtered (membrane filter with pore size of 0.45 μm; Schleicher & Schüll, Dassel, Germany). Each filter was placed on TTC agar (Oxoid GmbH, Wesel, Germany), Pseudomonas CN agar (Oxoid) and Slanetz-Bartley agar (Oxoid), and incubated at 36°C for five days.

For detection of *Legionella spp*., 1000 ml were filtered for better sensitivity. The filters were temporarily covered with HCl/KCl buffer (pH 2.2), incubated for 5 min and finally flushed with 10 ml sterile distilled water. The filters were placed onto selective agar (GVPC agar, Oxoid, Wesel, Germany) and incubated at 36°C for 1 week. Verification of visually suspicious colonies was performed by Legionella-latex test (Oxoid) and subculturing onto columbia agar (Oxoid) [19].

For detection of moulds, 100 ml of water were filtered (0.45 μm). The filters were placed on 4% Sabouraud glucose agar (Oxoid), incubated for 3 days at 37°C and afterwards for 4 days at 22°C. The grown moulds were identified microscopically after scotch taping the aerial mycelium and staining with lactophenol-cotton blue (Merck Darmstadt Germany) on slides (400-fold magnification).

## Results

### Trial 1

After one week, all samples fulfilled the recommendations with total cfu < 100/ml at 22°C and 36°C [17], and no pathogens/100 ml (data not shown) [2,16]. After the first reprocessing (end of 2nd week), one filter sample yielded mucilaginous colonies, i.e., *P. stutzeri *(7 cfu/ml), which was also cultured from the sludge samples of all filters together with a mucilaginous, aerobic, spore-forming bacillus. After 2 cycles of reprocessing (end of 3rd week) *P. stutzeri *was cultivated from the water samples of all filters (>> 100 cfu/ml). Therefore, the trial was stopped and reprocessing was completely changed.

### Trial 2

After the 7 weekly check periods, because all filters provided the required water quality, samples were taken once a month for 6 months with continous weekly filter change. For the whole trial, the recommended water quality was maintained (Table 3).

**Table 3:** Microbiological results with weekly (1–7) and monthly (11–31) test intervals in trial 2

**Filter No.**	**Parameter**	**Sampling week**												
		**1**	**2**	**3**	**4**	**5**	**6**	**7**	**11**	**15**	**19**	**23**	**27**	**31**
1	Total bacteria (cfu/ml) (22°C/36°C)	6/2	2/4	0/0	1/1	11/23	3/7	3/1	0/1	0/1	0/2	0/0	29/72	5/8
2		2/0	21/3	1/0	2/3	32/16	2/3	1/1	2/0	0/0	10/98	21/30	10/13	20/18
3		0/3	3/5	4/0	5/7	6/10	0/8	1/1	0/0	0/10	45/70	0/1	1/6	1/3
4		3/2	0/0	0/2	11/4	6/25	0/2	1/2	0/0	0/0	6/3	1/0	12/10	2/0
5		2/0	0/1	1/1	10/12	8/4	7/8	0/1	5/0	5/0	2/4	5/0	21/13	0/1
6		1/1	1/1	0/2	0/10	9/11	3/1	4/3	0/0	8/3	9/0	1/0	8/18	1/0
1–6	Pathogens* (cfu/100 ml)	0	0	0	0	0	0	0	0	0	0	0	0	0
1–6	*Legionella spp*.(cfu/1000 ml)	0	0	0	0	0	0	0	0	0	0	0	0	0
results without filter (unfiltered water load)	*Legionella pneumophila serogroup 1*	1	10	0	0	500	800	944	370	0	270	22	0	173
	*P. aeruginosa*	0	0	0	0	0	0	0	0	20	0	0	0	0
	Total bacteria (cfu/ml)	2/3	4/207	0/6	35/18	24/76	17/26	7/8	0/0	138/14	8/5	12/0	9/22	6/2

* *P. aeruginosa, E. coli*, coli-like, *faecal enterococci*

In the control (unfiltered water), in 9 of 13 samples *L. pneumophila serogroup 1*, and in 1 of 13 samples, *P. aeruginosa *were cultured. For both pathogens, it was possible to eliminate the bacterial contamination at all filter outlets completely (100%). In 2 of 13 samples, the total bacteria in unfiltered water surpassed 100 cfu/ml (Table 3). In none of the filter samples could ≥ 100 cfu/ml be cultured. The mean cfu/ml of unfiltered water was reduced from 20 ± 35.4 cfu/ml to 5 ± 7.5 cfu/ml at 22°C and from 30 ± 54.7 cfu/ml to 7 ± 16.0 cfu/ml at 36°C, which represents a reduction of about 25% for each temperature (data not shown).

The comparison of the sensitivity of the two culture temperatures – 22°C and 36°C – showed nearly identical results of 0 cfu/ml in 22 and 21 samples, resp., 1–10 cfu/ml in 46 and 44 samples, resp., and 11–100 cfu/ml in 10 and 13 samples, resp. (raw data Table 3). As a consequence, we decided to cultivate only at 22°C in trial 3.

### Trial 3

Over the test period of three months, no pathogens were cultured. During the test period, the reduction efficacy against *Legionella ssp*. and *P. aeruginosa *was 100%, as shown by parallel testing of unfiltered water with a mean load of 480 cfu/1000 ml of *Legionella pneumophila sero group 1 *and 120 cfu/100 ml of *P. aeruginosa*. The mean cfu of total bacteria at 22°C ranged from 1.6 to 28.9 in the filtered water (Table 4). The calculated mean reduction ranged between 25 and 95%.

**Table 4:** Total bacteria at 22°C (mean cfu/ml) from weekly testing of filters changed monthly (each n = 18, trial 3)

Month	cfu/ml				
	
	week 1	week 2	week 3	week 4	control before changing
1^st^	12.4	13.4	23.1	14.0	540
2^nd^	3.8	1.6	8.8	28.9	273
3^rd^	5.6	8.8	24.7	18.1	135

All but three samples (116, 167 and 187 cfu/ml) fulfilled the drinking water recommendations (< 100 cfu/ml). The three corresponding filters showed visible external contamination (mineral stains from splashed water) and provided < 100 cfu/ml after cleansing disinfection.

### Trial 4

Over the complete test period of 8 weeks, the reduction efficacy against *Legionella spp*. was 100%. On one shower filter, one cfu of *Pseudomonas aeruginosa*/100 ml was found in the third sample set; all other filter samples at all times showed 100% reduction. For total bacteria at 22°C, all samples of the filtered water yielded < 100 cfu/ml, as they also did at 36°C, with the exception of 5 samples (between 119 and 198 cfu/ml). The mean of total bacteria at 22°C and 36°C ranged from 3.8 to 14.4 and 14.5 to 34.7 cfu/ml, resp. (Table 5). The calculated mean reduction reached 99 and 94.5%, resp. Surprisingly, the obtained mean amount of moulds (*Aspergillus *and *Penicillium spp*.) decreased from 1.0 to 0.1 to finally 0 after 8 weeks (mean mould load in the unfiltered water of *Aspergillus spp*., *Penicillium spp*. and *Pseudallescheria boydii *was 5.2 cfu/100 ml) (Table 5).

**Table 5:** Total bacteria and *P. aeruginosa *(mean cfu/ml or/100 ml) of filters with operating time of 8 weeks (each n = 18 samples, trial 4)

Test parameter	cfu/ml			
	
	week 2	week 4	week 8	control before changing (final reduction %)
total bacteria 22°C	14.4	3.8	6.3	607 (99)
total bacteria 36°C	29.4	14.5	34.7	635 (94.5)
*P. aeruginosa*	0	0	0.06*	344 (97.5)
Moulds	1	0.1	0	5.2 (100)

* only 1 filter (shower) exhibited 1 cfu/100 ml

In trials 3 and 4, the unfiltered water contamination did not quantitatively differ from trial 2, with intermittent presence of *L. pneumophila serogroup 1 *and *P. aeruginosa *(data not shown).

### Comparative evaluation of filter efficacy in trials 2–4

**Figure 1:**
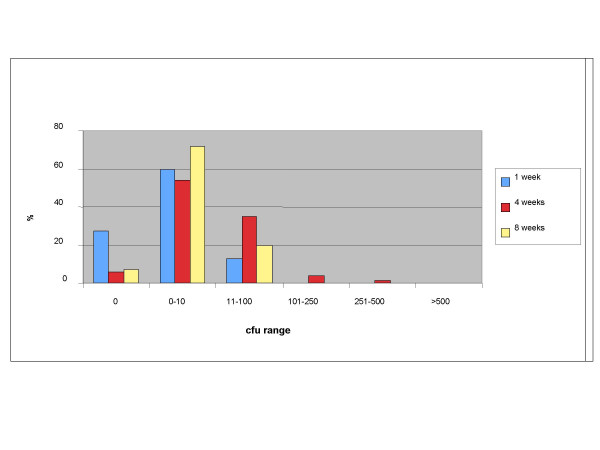
Percentage of colony forming units (22°C) at increasing operation times (results of trials 2–4).

**Figure 2:**
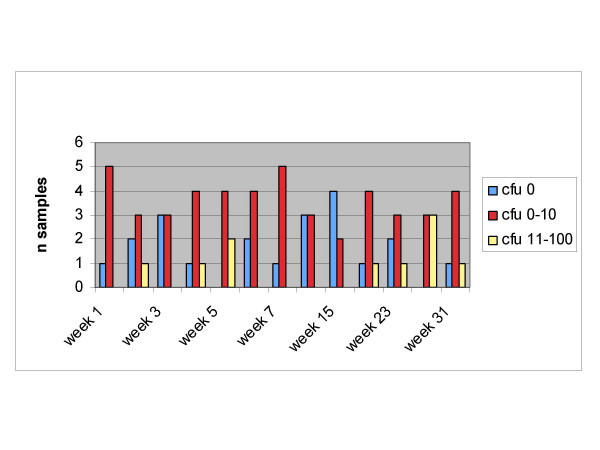
Longitudinal stability of the filter efficacy for total bacteria counts in trial 2 (22°C, 31 weeks, 6 tap filters).

The fact that the filter material and construction itself was identical in each filter type (only the encasements differed) allowed a direct comparison of the filters. Overall, the filtered water fulfilled the requirements of absence of pathogens over the different operating times. The number of cfu did not increase with longer operating times from 1 week to 8 weeks (Figure 1). For stable routine use, it is important that the filter efficacy shown for 7 days operating time could be demonstrated for 31 weeks (Figure 2).

The filter efficacy of the 3 filter types did not differ significantly regarding total bacteria, when cultivated at 22°C (table 6), which could be explained by retrograde contamination during the practical use. As no reprocessed filter exhibited 100% filter efficacy, as proven in the final quality control after each reprocessing, any contaminants in the filter samples could be explained by retrograde contamination during use. Therefore, we implemented specifying training for the cleaning staff and users. At every filter site, a warning sign with short instructions was posted.

**Table 6:** Filter efficacy (mean cfu/ml at 22°C) of filter type 1–3

**Operating time (weeks)**	**trial 2/filter type 1 (n samples)**	**trial 3 (filter type 2) (n samples)**	**trial 4 (filter type 3) (n samples)**
1	3.1 ± 5.9 (78)	7.3 ± 8.8 (54)	14.4 ± 17.5 (18)
4	-	20.0 ± 27.4 (54)	3.8 ± 4.6 (18)
8	-	-	6.3 ± 11.5 (18)

## Discussion

Following the recommendations of the Robert Koch Institute [20], the reprocessing of point-of-use water filters as semi-critical medical devices should include cleaning and thermic disinfection, preferably by washer/disinfectors that can provide validated and quality-controlled processing. The validity of this recommendation is supported by our study. As long as reusable filters are not processed in this manner, safe drinking water quality cannot be guaranteed. Tap water bacteria, especially the mucilaginous contaminant *P. stutzeri*, accumulated as a biofilm in the filters, as demonstrated by the insufficiency of simple manual reprocessing by boiling and flushing. A key to effective disinfection is removing the bio load accumulated on the filter membranes. This could be only achieved by strong chemical cleaning, followed by flushing and thermic disinfection in a washer/disinfector. Wendt et al. [21] showed that *Legionella*-free water cannot be achieved by using non-flushable POU filters, although the filters were automatically reprocessed in an autoclave. Vonberg et al. [22] also tested the reusable and flushable Germlyser filters with manual reprocessing and found pathogen-free water, but unacceptably high total germ counts. In the light of our results with reprocessing conducted via washer/disinfector, we conclude that this contamination was caused by the described insufficient manual reprocessing and not by recontamination as a consequence of inadequate handling, as postulated by the authors [22].

Over the entire study period, the 18 reprocessed filters provided pathogen-free water in accordance with the WHO guidelines, and additionally, total bacteria counts of < 100 cfu/ml, complying with German drinking water standards. The results of trials 3 and 4 show that the operating time can be extended up to 4 or 8 weeks, but as a consequence of sporadic contamination of the outer filter encasement, we recommend complete filter encasement disinfection by wiping with an alcohol-based product at least weekly (in high risk areas, both daily and directly after visible contamination), which proved to be successful in routine use. In high risk areas of our hospital, we decided to routinely use filters for 4 weeks, which normally corresponds with the length of hospitalisation. For outlets which are only occasionally used (e.g., for the birth bathtub), 8 weeks are safe. This management protocol has been implemented as part of our water safety plan for routine clinical use [23]. After reprocessing in a washer/disinfector, the filters are usable for two years. This data was generated in our neonatology intensive care department (13 filter sites), with weekly changing of filters and continuous monitoring of filter safety after each reprocessing, in keeping with the manufacturer's instructions. The manufacturer requests a leakage test after each reprocessing of a filter during its service life (52 reprocessing are guaranteed).

## Conclusion

As a consequence of our study, the manufacturer now recommends changing filters after 4 weeks of use in high risk areas and after 8 weeks in moderate infectious risk areas, together with routine, weekly alcohol-based surface disinfection and additionally in case of visible contamination.

## Competing interests

The author(s) declare that they have no competing interests.

## Authors' contributions

The study was carried out by GeDä, AK, CS, NR, WHK and GoDö. The manuscript was written by GeDä, AK and WHK. All authors read and approved the final manuscript.

## Pre-publication history

The pre-publication history for this paper can be accessed here:


